# Ethylene is all around

**DOI:** 10.3389/fpls.2015.00076

**Published:** 2015-02-12

**Authors:** Domenico De Martinis, Tomotsugu Koyama, Caren Chang

**Affiliations:** ^1^ENEA Italian National Agency for New Technologies, Energy and Sustainable Economic DevelopmentRome, Italy; ^2^Suntory Foundation for Life SciencesOsaka, Japan; ^3^Department of Cell Biology and Molecular Genetics, University of MarylandCollege Park, MD, USA

**Keywords:** ethylene, plant biology, plant developmental biology, fruit ripening, plant reproduction, plant senescence, plant stress biology, plant-microbe interactions

The small and simple gaseous molecule ethylene (C_2_H_4_) is usually associated with ripening and senescence events in plants. Its effects have been known for ages (literally), and in the last century there have been some major breakthroughs in ethylene biology. These breakthroughs include the discovery that the molecule that hastened the ripening of fruits and caused growth distortions in plants was indeed ethylene (early 1900's), the demonstration that while generally associated with leaking gas mains, ethylene is produced by plants themselves (1930) and finally, the description of the ethylene biosynthesis pathway by Shang Fa Yang (the “Yang cycle” 1979) (Bradford, 2008).

Yet, it was at the end of the Twentieth century that research on ethylene received massive attention with the understanding of how the ethylene chemical signal is perceived and processed by plants via complex mechanisms and that almost all physiological events in plants are influenced by ethylene.

This is probably why ethylene research in plant science is so broad and active; biosynthesis, perception, signal transduction and mode of action indeed keep scientists from different disciplines very busy.

This activity and breadth in our Frontiers research topic are clearly in evidence with the involvement of scientists describing many of these aspects of ethylene biology.

Metabolism is reviewed by Rodrigues and colleagues (Shedding light on ethylene metabolism in higher plants) describing both ethylene biosynthesis and the mechanisms controlling it, as well as the influence of light on ethylene evolution.

Ethylene biosynthesis and ripening is approached by Hoogstrate and colleagues, analyzing a single ACC synthase gene, *ACS4*, and demonstrating how *ACS4* is necessary for the normal progression of tomato fruit ripening and how mutation of this gene (out 12 ACS genes in the tomato genome) may provide a useful means for altering ripening traits.

The effects of ethylene on the fruit *metabolome* and the resulting fruit quality are not fully understood. In their study, Sobolev et al. approach the issue, showing that ripening-associated metabolic changes are both ethylene dependent and independent, and that the fruit *metabolome* is under the control of multiple regulators.

The role of ACS is also described in the model plant, poplar. In their study, Plett et al. describe how ethylene affects the important agronomic trait of plant height. The study also describes the effect of ethylene on leaf senescence, an important biological event that is more broadly described in the reviews by Tomotsugu Koyama and Kim et al. in terms of physiological events and molecular regulation.

The ethylene precursor, ACC itself, also serves as a substrate for several other derivatives. Van de Poel and Van Der Straeten describe the potential role of ACC as more than just the precursor of ethylene but as a means for plants to quickly communicate between organs, whenever ethylene diffusion is too slow.

Besides ripening, numerous other events are regulated by ethylene. Bianka Steffens describes flooding, one of the main abiotic stresses resulting in crop yield loss, a worldwide problem in different landscapes. Some crop species endure soil waterlogging for some hours, others for days or months. Flooding can be associated with water quality changes (salinity, pH, and heavy metal concentration). One metabolic response is the induction of ethylene production and reactive oxygen species (ROS) that together play an important role in mediating numerous specific growth or cell death responses.

Ethylene perception is described in the original research paper by Wilson et al. In their paper, the authors also describe a physiological effect of loss of ethylene perception that reduces the effect of far-red light and darkness on seed germination.

A possible mode of action of signal translation is addressed in the perspective by Zhang et al. and the review by Cho and Yoo, while ethylene effects on germination are reviewed by Corbineau et al.

Last but not least, the important role of ethylene in plant-microbe interaction with the biotech relevant *Agrobacterium* is reviewed by Nonaka and Ezura.

The breadth of participation has been as broad as the topic itself. This research topic arrives after approximately 10 months from the initiative of Frontiers in Plant Science to invite us to serve as guest associate editors. During those months we contacted about one hundred scientists worldwide including established experts in the fields as well as “young guns.” We eventually published 13 articles involving authors from Europe, North and South America, and Asia. The result is an issue with a broad view that matches and complements the others topics in the “signaling” area.

In this topic, the fascinating biology of ethylene has led us to approach, in modern terms, questions posed about 100 years ago. How many more questions will arise? It must be noted that ethylene is also massively produced and widely used in chemical industry and can even be released by humans (Cristescu et al., 2014).

Chemistry and Medicine other than Biology are therefore the domain of research and development for ethylene; and the more we study, the more we discover that ethylene, indeed, is all around.

## Conflict of interest statement

The authors declare that the research was conducted in the absence of any commercial or financial relationships that could be construed as a potential conflict of interest.
